# Supplementary material for: mTOR Inhibitors Modulate the Biological Nature of TGF-β2-Treated or -Untreated Human Trabecular Meshwork Cells in Different Manners
Source: Biomedicines. 2024 Nov 14;12(11):2604. doi: 10.3390/biomedicines12112604 (PMC11591778; doi:10.3390/biomedicines12112604)
Supplement: Supplementary file 1 [file biomedicines-12-02604-s001.zip › biomedicines-3278619-supplementary.pdf]

Supplemental Table S1 The sequences of primers and probes used in this study.

		Sequence	Exon Location	RefSeq Number	Product Length (bp)
human RPLP0*1	Probe	5'-/56-FAM/CCCTGTCTT/ZEN/CCCTGGGCATCAC/3IABkFQ/-3'	2-3	NM_001002	143
	Primer2	5'-TCGTCTTTAAACCCTGCGTG-3'			
	Primer1	5'-TGTCTGCTCCCACAATGAAAC-3'			
human COL1A1*1	Probe	5'-/56-FAM/TCGAGGGCC/ZEN/AAGACGAAGACATC/3IABkFQ/-3'	1-2	NM_000088	115
	Primer2	5'-GACATGTTCAGCTTTGTGGAC-3'			
	Primer1	5'-TTCTGTACGCAGGTGATTGG-3'			
human COL4A1*1	Probe	5′-/56-FAM/TCATACAGA/ZEN/CTTGGCAGCGGCT/3IABkFQ/-3′	51-52	NM_001845	142
	Primer2	5′-AGAGAGGAGCGAGATGTTCA-3′			
	Primer1	5′-TGAGTCAGGCTTCATTATGTTCT-3′			
human COL6A1*1	Probe	5′-/56-FAM/CAGGTTTCG/ZEN/GTCACAGCGGTAGT/3IABkFQ/-3′	2-3	NM_001848	114
	Primer2	5'-CCTCGTGGACAAAGTCAAGT-3'			
	Primer1	5'-GTGAGGCCTTGGATGATCTC-3'			
human FN1*1	Probe	5′-/56-FAM/TACAGCTTA/ZEN/TTCTCCCTCGCCCAG/3IABkFQ/-3′	3-4	NM_212482	129
	Primer2	5'-CGTCCTAAAGACTCCATGATCTG-3'			
	Primer1	5'-ACCAATCTTGTAGGACTGACC-3'			
human αSMA*1	Probe	5'-/56-FAM/AGACCCTGT/ZEN/TCCAGCCATCCTTC/3IABkFQ/-3'	8-9	NM_001613	105
	Primer2	5'-AGAGTTACGAGTTGCCTGATG-3'			
	Primer1	5'-CTGTTGTAGGTGGTTTCATGGA-3'			
